# The Hidden Truths of Fungal Virulence and Adaptation on Hosts: Unraveling the Conditional Dispensability of Minichromosomes in the Hemibiotrophic *Colletotrichum* Pathogens

**DOI:** 10.3390/ijms25010198

**Published:** 2023-12-22

**Authors:** Vijai Bhadauria, Manyu Zhang, Wendi Ma, Jun Yang, Wensheng Zhao, You-Liang Peng

**Affiliations:** 1 Department of Plant Pathology, College of Plant Protection, China Agricultural University, Beijing 100193, China; s20213192958@cau.edu.cn (M.Z.); 18811305188@163.com (W.M.); yangj@cau.edu.cn (J.Y.); mppzhaws@cau.edu.cn (W.Z.); pengyl@cau.edu.cn (Y.-L.P.); 2 The Ministry of Agriculture and Rural Affairs for Key Laboratory of Crop Pest Monitoring and Green Control, College of Plant Protection, China Agricultural University, Beijing 100193, China

**Keywords:** anthracnose, conditionally dispensable chromosomes, fungal virulence/pathogenicity, fungal growth and development, CRISPR/Cas9-based minichromosome editing

## Abstract

*Colletotrichum* spp. are ascomycete fungi and cause anthracnose disease in numerous crops of economic significance. The genomes of these fungi are distributed among ten core chromosomes and two to three minichromosomes. While the core chromosomes regulate fungal growth, development and virulence, the extent to which the minichromosomes are involved in these processes is still uncertain. Here, we discuss the minichromosomes of three hemibiotrophic *Colletotrichum* pathogens, i.e., *C. graminicola*, *C. higginsianum* and *C. lentis*. These minichromosomes are typically less than one megabase in length, characterized by containing higher repetitive DNA elements, lower GC content, higher frequency of repeat-induced point mutations (RIPMs) and sparse gene distribution. Molecular genetics and functional analyses have revealed that these pathogens harbor one conditionally dispensable minichromosome, which is dispensable for fungal growth and development but indispensable for fungal virulence on hosts. They appear to be strain-specific innovations and are highly compartmentalized into AT-rich and GC-rich blocks, resulting from RIPMs, which may help protect the conditionally dispensable minichromosomes from erosion of already scarce genes, thereby helping the *Colletotrichum* pathogens maintain adaptability on hosts. Overall, understanding the mechanisms underlying the conditional dispensability of these minichromosomes could lead to new strategies for controlling anthracnose disease in crops.

## 1. Introduction

*Colletotrichum* spp. are ascomycete fungi and represent an economically significant group of pathogens of numerous crops worldwide, resulting in severe yield losses. The species include, but are not limited to, *C. graminicola*, *C. lentis* and *C. higginsianum*. *C*. *graminicola* (Ces.) G.W. Wilson causes the anthracnose leaf blight and stalk rot disease in maize (*Zea mays* L.), which is one of the top diseases affecting grain/silage production in maize-growing regions of the world, especially in the USA and Canada. From 2016 to 2019, the USA and Canada experienced a 0.21% annual yield reduction (3.03 million metric tons) due to anthracnose, resulting in a direct financial loss of USD 579.3 million [1]. *C*. *lentis* Damm causes anthracnose disease in lentils, which is the most destructive disease of lentils in Canada. In epidemic years, grain yield loss can reach up to 70% [2], which is equivalent to a direct financial loss of around USD 300 per ha for a lentil producer. *C. higginsianum* Sacc. infects cruciferous crops, such as *B. rapa* ssp. *pekinensis* (Chinese cabbage), *Brassica napus* (rapeseed), *B. rapa* ssp. *parachinensis* (Chinese flowering cabbage) and *B. rapa* ssp. *chinensis* (bok-choi), and the model plant species *Arabidopsis thaliana* [3,4,5].

These *Colletotrichum* spp. employ a hemibiotrophic infection strategy (biotrophy-to-necrotrophy or sequential biotrophy) to infect and subsequently colonize the invaded tissues. Fungal infection is initiated by single-celled asexual spores, called conidia, which, upon landing on the plant surface (e.g., leaves, leaf sheathes, leaf whorls and/or stalk rinds), germinate to form regular or irregular dome-shaped appressoria. A narrow penetration peg emanating from the contact point of the appressorium with the plant surface pierces the epidermal cuticle and cell wall and elaborates the infection vesicle, which then expands to form thick biotrophic hyphae. In the case of *C. lentis* and *C. higginsianum*, the biotrophic hyphae (primary hyphae) are confined to the first invaded epidermal cells, and a complete switch to necrotrophy ensues thereafter, where thin necrotrophic hyphae differentiate from the biotrophic hyphae that kill and macerate the colonized tissues. However, *C. graminicola* exploits a unique hemibiotrophic infection strategy called sequential biotrophy [6], where the biotrophic hyphae of C. *graminicola* continuously proliferate well beyond the first infected epidermal cells and, therefore, the edges of infection courts remain biotrophic while the centers thereof become necrotrophic characterized secondary hyphae. Water-soaked lesions appear at the infection court within a few days of infection, and acervuli with black setae and conidia embedded in the salmon-pink-colored gelatinous matrix are produced in the anthracnose lesions within a week. These conidia are dispersed by splashing and blowing raindrops to infect neighboring healthy plants.

How these *Colletotrichum* spp. modulate their virulence/pathogenicity on their hosts have been the subject of numerous studies, which were primarily focused on pinpointing single genes located on core chromosomes through phenotyping of their knockout mutants on hosts. Until recently, delimiting the role of chromosomes was an arduous task, primarily concomitant with the lack of chromosome-level genome assemblies and functional analysis approaches. However, with the advent of second- and third-generation sequencing, and highly efficient genome assemblers, fungal genomes are being sequenced at the chromosome level. More recently, a gln-tRNA-based CRISPR/Cas9 minichromosome deletion system was developed, enabling the determination of the biological role of large genomic regions, including minichromosomes, by generating their knockout mutants and phenotyping them for fungal growth and development and virulence/pathogenicity on hosts [7].

Whole-genome sequencing of the three *Colletotrichum* spp. (*C. graminicola*, *C. lentis* and *C. higginsianum*) reveals that they carry ten core chromosomes (>3 Mb in length) and two to three minichromosomes (<1 Mb in length) [7,8,9,10,11]. Molecular genetics and functional analyses reveal that one of the minichromosomes shows conditional dispensability, i.e., this minichromosome is dispensable for fungal growth and development but modulates fungal virulence on hosts [7,9,11]. Here, we review the minichromosomes of three *Colletotrichum* spp. (*C. graminicola*, *C. lentis* and *C. higginsianum*), highlighting the genic and genomic diversity underlying the virulence/pathogenicity and adaptation of these pathogens on hosts.

## 2. *Colletotrichum graminicola*

**Figure 1:**
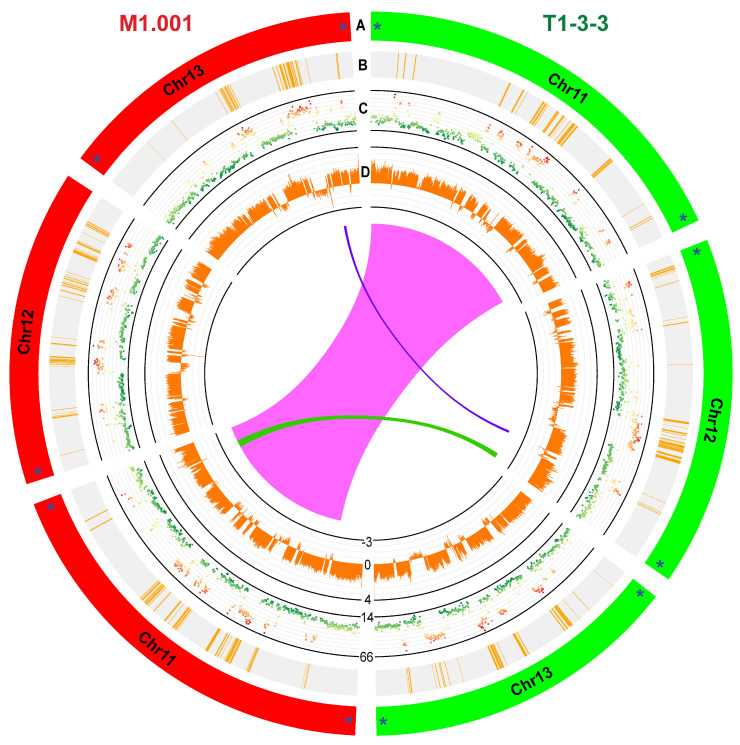
Circos plot displaying variability between minichromosomes (Chr11 through Chr13) of the two reference strains (T1-3-3 and M1.001) of the maize anthracnose fungus *Colletotrichum graminicola*. Tracks: (A) minichromosomes (T1-3-3-Chr11 (7.14 Mb), T1-3-3-Chr12 (6.19 Mb) and T1-3-3-Chr13 (5.62 Mb); M1.001-Chr11 (7.29 Mb), M1.001-Chr12 (5.59 Mb) and M1.001-Chr13 (5.51 Mb)); (B) gene models; (C) GC content; and (D) composite index values (CIVs) calculated as (TpA/ApT)–(CpA + TpG/ApC + GpT). CIVs surpassing 0 signal the presence of repeat-induced point mutation (RIPM) activity. For RIPM analyses, a window size of 1000 bp and a step size of 500 bp were utilized. RIP mutations are restricted to regions of the minichromosomes that are low in GC content and devoid of genes. Asterisks indicate telomeric ends. Links represent syntenic regions between the minichromosomes of T1-3-3 and M1.001.

The reference genomes of the two strains (M1.001 (57.43 Mb) and T1-3-3 (60.98 Mb)) of *C. graminicola* have been recently sequenced at the chromosome level using second- and third-generation sequencing platforms [7,10]. M1.001 and T1-3-3 were isolated from infected maize leaves, respectively, in Missouri, the USA, and Hebei, China [12,13]. Both strains carry three minichromosomes (Chr11–Chr13) and ten core chromosomes. The gapless genome assemblies of both strains contain three telomere-to-telomere-sequenced minichromosomes, except M1.001-Chr12, which lacks the 3′ telomeres (Figure 1). The minichromosomes of both strains are less than one megabase in length; however, they exhibit length polymorphisms, e.g., T1-3-3-Chr11 is 15,291 bp shorter than M1.001-Chr11, whereas T1-3-3-Chr12 and T1-3-3-Chr12 are 59,458 and 11,260 bp larger than M1.001-Chr12 and M1.001-Chr13, respectively (Table 1).

Repeat-induced point mutations (RIPMs) are a genome defense mechanism that has evolved exclusively in fungi and occur during sexual reproduction, helping to preserve genome integrity from repetitive DNA elements, such as tandem (e.g., satellites and simple sequence repeats) and interspersed repetitive DNA elements (e.g., retroelements (short and long interspersed elements)), and DNA transposons [14,15,16]. They cause cytosine to thymine transitions in duplicated and repeated sequences [14]. Such mutations are often coupled with epigenetic silencing of the RIP-affected genomic regions through methylation of the remainder of cytosine residues in the duplicated and repeated sequences in order to ameliorate the deleterious effects of repetitive DNA elements, especially DNA transposons. Generally, the genomic regions not targeted by RIPMs are GC-rich, whereas those affected are AT-rich. Three-quarters of the minichromosomes of the T1-3-3 and M1.001 strains are affected by RIPMs, e.g., M1.001-Chr1 (77.38%), -Chr12 (74.44%) and -Chr13 (75.68%), and T1-3-3-Chr11 (80.97%), -Chr12 (71.65%) and -Chr13 (75.36%) (Table 1). As a result of extensive RIPMs, the two strains carry highly compartmentalized minichromosomes, with 75% of their sequences occupied by AT (ApT and TpA)-rich blocks and 25% by GC-rich blocks. The AT-rich blocks are likely to prevent the proliferation of DNA transposons in these minichromosomes. The minichromosomes of two strains, therefore, carry lower GC content and higher repetitive DNA content, e.g., M1.001-Chr1 (29.90% GC content; 10.59% repetitive DNA elements), -Chr12 (30.10% GC content; 10.78% repetitive DNA elements) and -Chr13 (30.31% GC content; 8.86% repetitive DNA elements), and T1-3-3-Chr11 (29.10% GC content; 11.14% repetitive DNA elements), -Chr12 (31.24% GC content; 11.11% repetitive DNA elements) and -Chr13 (30.74% GC content; 12.13% repetitive DNA elements) (Table 1). Although RIPM-affected regions have no known biological functions except for acting as a countermeasure against repetitive DNA elements, they may help protect the genic regions of the minichromosomes by stabilizing their non-genic flanking regions, thereby maintaining the plasticity and adaptability of the pathogens in terms of their virulence/pathogenicity on hosts.

The T1-3-3 and M1.001 strains share only Chr11, whereas Chr12 and Chr13 are strain-specific innovations. These minichromosomes lack virulence-/pathogenicity-related genes, such as those coding for effectors and secondary metabolite enzymes, except for T1.3.3-Chr12, which harbors an effector candidate gene *GME10857*, whose expression, however, is repressed during maize infection (log2fc ≤ 1.5, *p* < 0.01). In addition, *GME10857* is not required for fungal growth and development, or *C. graminicola* virulence on maize.

T1-3-3-Chr11 (0.71 Mb) and M1.001-Chr11 (0.73 Mb) carry, respectively, 32 and 43 genes, 25 of which are homologous genes (Supplementary Table S1) [7,10]. Twenty-four of the twenty-five genes encode proteins of unknown functions, whereas one (GME6619 (T1-3-3) and CGRA01v4_15035 (M1.001)) codes for ubiquitin-like protein-specific protease 1 (Ulp1). SUMOylation is a reversible post-translational modification in which SUMOs (small ubiquitin-like modifiers) are covalently attached to lysine residues in the substrate proteins, and this process is catalyzed by SUMO-activating enzymes (E1), SUMO-conjugating enzymes (E2), SUMO ligases (E3) and SUMO proteases [17,18]. In fungal pathogens, SUMOylation contributes to infection-related fungal development and virulence, as well as stress responses [19]. Ulp1 is a SUMO protease that deSUMOylates nuclear and cytoplasmic proteins [20]. *GME6619* is induced (log2fc 5.5, *p* < 0.01) during maize infection at 72 hpi, where the infection court is replete with primary and secondary hyphae. The mutant lacking *GME6619*, however, does not show any defect in colony growth and conidiogenesis or virulence on the maize inbred line B73. T1-3-3 carries an additional tandem *Ulp1* gene paralog *GME6620*, which is repressed during maize infection. The majority of non-homologous genes in Chr12 also code for proteins of unknown functions, except for *GME6622* (T1-3-3), and *CGRA01v4_15032*, *CGRA01v4_15038*, *CGRA01v4_15051* and *CGRA01v4_15054* (M1.001). *GME6622* codes for exo-1,3- β -D-glucanase. Fungal pathogens generate β-1,3-glucans for cell wall remodeling during plant infection, which, however can trigger pattern-triggered immunity (PTI). Recently, a CAZyme exo-β-1,3-glucanase (Ebg1) from the rice blast fungus *Magnaporthe oryzae* is reported to hydrolyze β-1,3-glucan, thereby suppressing PTI [21]. *CGRA01v4_15032*, *CGRA01v4_15038* and *CGRA01v4_15051* encode unc-44/ankyrin proteins. UNC-44/ankyrin plays a crucial role in the TMC-1 mechanotransduction channel complex situated in the sensory cilia of the mechanoreceptor neuron of *Caenorhabditis elegans* and is essential for proper axonal guidance [22,23]. However, its role in fungal pathogens remains unknown. T1-3-3-Chr11 carries a strain-specific gene *GME6639*, which, however, does not express in vegetative mycelia or infected maize tissues. CGRA*01v4_15054* codes for a phosphorylase superfamily protein [10]. M1.001-Chr12 possesses two strain-specific genes (*CGRA01v4_15014* and *CGRA01v4_15026*). Interestingly, only four (*GME6613*, *GME6617*, *GME6618* and *GME6619*) encoding proteins of unknown functions of the thirty-two genes in T1-3-3-Chr11 are induced during plant infection (log2fc ≥ 1.5, *p* < 0.01) [7]. These genes are, however, not required for fungal growth and development or the *C. graminicola* virulence on maize. Transcriptional repression of the genes in T1-3-3-Chr12 might be associated with the presence of seven miRNAs (four mi-598 and three mir-684) (Supplementary Table S2).

T1-3-3-Chr12 (0.62 Mb) and M1.001-Chr12 (0.56 Mb) carry 31 and 43 genes, respectively; none are homologous genes. Eight and six, respectively, of the T1-3-3-Chr12 and M1.001-Chr12 are strain-specific (Supplementary Tables S3 and S4). The remaining genes in T1-3-3-Chr12 encode proteins of unknown functions except for *GME10862*, which codes for LPXTG domain-containing protein. The LPXTG domain, in conjunction with a C-terminal sorting signal, enables the anchoring of surface proteins in the bacterial cell walls [24]; however, the presence of LPXTG domain-containing proteins in fungal pathogens has not been reported, let alone their role in these organisms. *GME10862* does not, however, express during vegetative growth and maize infection. The remaining genes in M1.001-Chr12 also code for proteins of unknown functions except for *CGRA01v4_15056*, *CGRA01v4_15083*, *CGRA01v4_15084*, *CGRA01v4_15085*, *CGRA01v4_15095*, *CGRA01v4_15096* and *CGRA01v4_15097*, which encode sentrin/sumo-specific protease, linoleate diol synthase, chloroperoxidase, VID27 cytoplasmic protein, BRO1-like domain-containing protein and methyltransferase type 11, respectively. Similar to Ulp1, sentrin/sumo-specific protease is a SUMO protease that deSUMOylates the substrate proteins by cleaving off SUMOs. Plant pathogenic ascomycete fungi secrete chloroperoxidases into hosts that catalyze the H_2_O_2_-dependent oxidation of Cl^−^ to hypochlorous acid [25,26,27,28]. A vanadium chloroperoxidase (MoVcpo) from *M. oryzae* induces reactive oxygen species (ROS; e.g., H_2_O_2_) in rice cells and appears to act as a pathogen-associated molecular pattern. In addition, *MoVcpo* is required for conidiogenesis, conidial germination, cell wall integrity, osmotic stress tolerance and *M. oryzae* virulence on rice [29]. BRO1-like domain-containing proteins are known to be involved in the fungal pH signaling pathway [30] and may be essential for the hemibiotrophic lifestyles of pathogens like *M. oryzae* and *Colletotrichum* spp., which require an alkaline host apoplastic environment during the biotrophic phase of infection and an acidic host environment during the necrotrophic phase of infection [31]. Interestingly, only two genes (*GME10839* and *GME10865*) encoding proteins of unknown functions in T1-3-3-Chr12 are induced during maize infection (log2fc ≥ 1.5, *p* < 0.01) [7]. However, these genes do not regulate colony growth, conidiogenesis or *C. graminicola* virulence on maize. Six of the eight T1-3-3-specific genes are not expressed, whereas the remaining *GME10852* and *GME10863* are repressed during maize infection. Transcriptional repression of the genes in T1-3-3-Chr12 might be concomitant with the presence of four miRNAs (mi-598) (Supplementary Table S2).

T1-3-3-Chr13 (0.56 Mb) and M1.001-Chr13 (0. 55 Mb) carry 26 and 35 genes, respectively; none of them are homologous genes. Six and ten, respectively, of the M1.001-Chr13 and T1-3-3-Chr13 are strain-specific (Supplementary Tables S5 and S6). The remaining genes in M1.001-Chr13 and T1-3-3-Chr13 code for proteins of unknown functions. Interestingly, only two genes (*GME10831* and *GME10833*) encoding proteins of unknown functions in T1-3-3-Chr13 are induced during maize infection (log2fc ≥ 1.5, *p* < 0.01) [7]. However, the mutants lacking these two genes are indistinguishable from the wild-type strain pertaining to fungal growth and development, and the *C. graminicola* virulence on maize. Five of the ten T1-3-3-specific genes are not expressed, whereas the remaining *GME10818* and *GME10819* are repressed during maize infection. Transcriptional repression of the genes in T1-3-3-Chr13 might be linked with four mi-598 that it carries.

A lack of effective molecular techniques to knock out fungal pathogen minichromosomes has hindered the delimitation of their biological functions. Until recently, targeted deletion of minichromosomes has not been attempted in any fungal species, including *Colletotrichum* spp., which are recalcitrant to genetic manipulations; this would have provided direct evidence of their role in fungal growth and development, and virulence/pathogenicity. Recently, we have developed a glutaminyl (gln)-tRNA-based CRISPR/Cas9 genome deletion system to functionally characterize the minichromosomes of T1-3-3 [7]. The system involves two types of vectors: pCas9-Cg_tRp-sgRNA carrying 20 bp minichromosome-specific protospacers and pCE-Zero-Hpt carrying the selectable marker gene *hygromycin phosphotransferase* (*Hpt*) flanked by the minichromosome-specific homology arms of 1000 to 2000 bp. In pCas9-Cg_tRp-sgRNA, a T1-3-3-specific gln-tRNA promoter efficiently drives the expression of sgRNAs. Upon the PEG/CaCl_2_-mediated co-transformation in the T1-3-3 protoplasts, the pCas9-Cg_tRp-sgRNA vectors generate multiple simultaneous DNA double-strand breaks (DSBs) across a targeted genomic region within a minichromosome, followed by homology-directed repair of DSBs with the homology arms. Using this system, functionally nullisomic mutants of Chr11, Chr12 and Chr3 of T1-3-3 were obtained. The Δ*Chr11*, Δ*Chr12* and Δ*Chr13* mutants lack 618.61, 498.69 and 393.98 Kb genomic regions of their respective minichromosomes, which carry 32, 31 and 26 genes, respectively. Phenotypic analysis of these mutants showed that Δ*Chr11*, Δ*Chr12* and Δ*Chr13* were indistinguishable from T1-3-3 in colony growth and conidiation and that only Δ*Chr12* was attenuated in virulence on the maize inbred line B73. This suggests that Chr12 is a conditionally dispensable minichromosome, which is dispensable for fungal growth and development but indispensable for fungal virulence on maize [7].

## 3. *Colletotrichum lentis*

The *C. lentis* isolates from lentil in Canada have been classified into two races (0 and 1) based on their reactions to a differential lentil cultivar CDC Robin. The race 0 isolates (e.g., CT-30) are fully pathogenic on CDC Robin (90–10%), whereas the race 1 isolates (e.g., CT-21) showed attenuated virulence (~15%) on the cultivar [9].

The reference genome of the *C. lentis* race 0 isolate CT-30 was sequenced using the second-generation Illumina sequencers (Illumina HiSeq 2000 and MiSeq), resulting in an assembly of 56.10 Mb distributed among 50 scaffolds. To assemble the genome at the chromosome level, an ascospore-derived population of 94 progeny isolates originating from a cross between CT-30, the race 1 isolate CT-21 and the parental isolates were resequenced on Illumina HiSeq 2000 at 19.08-fold genomic coverage. Mapping the resulting sequence reads onto the CT-30 genome yielded 14,132 high-quality single-nucleotide polymorphisms (SNPs), the most frequent genetic variations across the genomes. Genetic linkage mapping of these SNPs based on their genetic recombination resulted in 12 linkage groups, which were utilized to order and orientate 50 scaffolds into 12 chromosomes. Ten of the chromosomes are core chromosomes and >3 Mb in length, whereas two are minichromosomes (Chr11 and Chr12) [9]. Unlike minichromosomes, Chr11 is unusually large (1.52 Mb); Chr12 is 0.39 Mb in length. Unlike *C. graminicola*, the minichromosomes in *C. lentis* are less affected by RIPMS, e.g., Chr11 (9.21%) and Chr12 (8.02%); therefore, they carry higher GC contents, e.g., Chr11 (37.57%) and Chr12 (37.26%) (Table 1; Supplementary Tables S7 and S8).

Unlike *C. graminicola*, the *C. lentis* minichromosomes carry virulence-/pathogenicity-related genes, such as those coding for effectors, CAZymes and secondary metabolite enzymes. Chr11 carries 164 genes, 39 of which are unique to CT-30. Thirty-eight of the Chr11 genes code for proteins of unknown functions, whereas the remaining eighty-seven genes encode proteins, including nine effector candidates and four secondary metabolite backbone synthesis enzymes (SMBSEs). The *C. lentis* genome carries four genes, which code for NUDIX hydrolase domain-containing proteins. Of these, three were located on core chromosomes (scaffold1-552 (Chr9), scaffold12-9 and *scaffold12-65* (Chr7)) and one (*scaffold14-4*) on minichromosome Chr11. *scaffold12-65* is induced exclusively at the hemibiotrophic switch, where the necrotrophic hyphae start emanating from the biotrophic. Scaffold12-65 (aka ClNUDIX) induces a hypersensitive cell death response (HR) in *Nicotiana tabacum*. The *C. lentis* and *M. oryzae* strains overexpressing *ClNUDIX* trigger HR in the lentil cultivar Eston and the barley cultivar CDC silky, respectively. ClNUDIX initially accumulates along the cell periphery and later is taken up in endocytosis pits or early endosomes. The effector may hydrolyze the pyrophosphate bonds of inositol pyrophosphates (IP6 and IP7). The NUDIX motif within the NUDIX hydrolase domain specifically binds to IP6 and IP7, which in turn tether to clathrin-associated proteins, such as adaptor protein 2, a constituent of clathrin protein-coated endocytic pits and early endosomes that originate from the endocytic pits. The hydrolysis of inositol pyrophosphates by NUDIX hydrolase may dismantle the endocytosis pit or the early endosomes, resulting in the loss of plasma membrane integrity and causing non-specific HR due to the influx of extracellular proteins into cytoplasm. Since HR cell death coincides with the hemibiotrophic switch phase, it signals the pathogen to initiate the anthracnose-causing necrotrophic phase [32]. Like *scaffold12-65*, *scaffold14-4* is also exclusively induced during 48 hpi (log2fc 5.0, *p* < 0.01), corresponding to the hemibiotrophic switch phase of infection. These NUDIX hydrolase genes might have redundant functions in *C. lentis*. Silencing the NUDIX domains of these effectors may provide evidence for their direct involvement in the hemibiotrophic switch.

Interestingly, Chr11 carries two (*Pep1* (*scaffold14-51*) and *PDA1* (*scaffold14-146*)) of the six pea pathogenicity genes (*Peps*), which are known to be located on the conditionally dispensable minichromosome (1.60 Mb) of *Fusarium oxysporum* f. sp. *pisi*, which is a soilborne pathogen causing Fusarium wilt in peas. *PDA1* codes for pisatin demethylase, which detoxifies the phytoalexin pisatin produced as a defense response by pea [33]. *PDA1* is highly induced in *C. lentis* strain CT-30-infected CDC Robin leaf tissues (logfc 140, *p* < 0.01), whereas it shows marginal induction (logfc 1.2, *p* < 0.01) in *C. lentis* strain CT-21-infected CDC Robin leaf tissues [9]. In *F. oxysporum* f. sp. *pisi*, *Pep1* and *PDA1* are located 25 Kb apart, and in *C. lentis* 36.5 Kb apart. Lentil and pea are grown in rotation in Canada; therefore, it is plausible that Chr11 may be a horizontally transferred minichromosome.

Chr12 carries 38 genes, 10 of which are unique to CT-30. Fifteen of the Chr12 genes code for proteins of unknown functions, whereas the remaining 13 genes encode proteins of known functions, including polyketide synthases (PKS), non-ribosomal polyketide synthetases (NRPS), Ulp1, NB-ARC domain-containing protein, serine/threonine-protein kinase and conserved glycine-rich protein. The *C. lentis* genome contains 43 SMBSE-coding genes, 5 and 7 of which are located on Chr11 and 12, respectively. Six of the seven SMBSE-coding genes (*scaffold20-9, scaffold20-10, scaffold20-11, scaffold20-12, scaffold20-13* and *scaffold20-14*) encode NRPS, whereas *scaffold20-6* codes for PKS. Fungal pathogens produce a wide range of secondary metabolites, some of which are involved in fungal virulence/pathogenicity on hosts. There are four major groups of secondary metabolites: polyketides, bioactive peptides, alkaloids and terpenes [34]. NRPS and PKS are SMBSEs and catalyze the biosynthesis of bioactive peptides and polyketides, respectively.

To identify the genomic region controlling *C. lentis* virulence on lentil, the CT-30 x CT-21 ascospore-derived population (*n* = 94) was phenotyped for anthracnose severity on the Horsfall–Barratt scale. The severity scores were regressed on the genetic linkage map using composite interval mapping, resulting in the identification of a single quantitative trait locus (QTL; *qClVir-11*) located on minichromosome Chr11 modulating 85.23% of the virulence on lentil. *qClVir-11* is 0.84 Mb in length and contains 98 genes. Ten of these are induced during Eston (susceptible lentil cultivar) infection (log2fc > 2, *p* < 0.01), including *scaffold14-47* (unique to CT-30), *scaffold14-48* (unique to CT-30), *scaffold14-50* (hypothetical protein), *scaffold14-52* (hypothetical protein), *scaffold14-55* (hypothetical protein), *scaffold14-56* (subtilisin-like protease 2), *scaffold14-78* (unique to CT-30), *scaffold14- 83* (carbon-nitrogen hydrolase), *scaffold14-87* (mitochondrial chaperone bcs1), *scaffold14-129* (alcohol dehydrogenase 2) and *scaffold14-130* (nucleoside-diphosphate-sugar epimerase). Overall, the study by Bhadauria and colleagues [9] provided genetic evidence of the conditional dispensability of minichromosomes.

## 4. *Colletotrichum higginsianum*

Dallery and colleagues [8] recently resequenced the *C. higginsianum* strain IMI 349063A using the third-generation sequencer (Pacific Biosciences RS II) and aligned the resulting high-quality reads onto the optical map generated in the previous study [12]. The resulting 50.72 Mb genome is distributed among ten core chromosomes (average length > 4 Mb) and two minichromosomes (Chr11 (0.65 Mb) and Chr12 (0.60 Mb)). Similar to *C. lentis*, the *C. higginsianum* minichromosomes are less affected by RIPMs, e.g., Chr11 (8.89%) and Chr12 (15.38%), and are thus not highly compartmented into AT-rich and GC-rich blocks. As a result, they carry higher GC and gene contents, i.e., Chr11 (49.35%; 138 genes) and Chr12 (47.20%; 133 genes) (Table 1; Supplementary Tables S9 and S10).

Chr11 contains 133 genes, 98 of which code for hypothetical proteins, whereas the remaining 35 encode proteins of known functions, including 7 effector candidates and 1 each of CAZyme (glycosyl hydrolase family 92) and SMBSE (PKS-NRPS hybrid enzyme). One of the effector candidate genes*, CH63R_14535*, encodes a LysM domain-containing protein. Fungal LysM domain-containing proteins are small secreted proteins that bind to chitin oligosaccharides during plant–pathogen interactions, thereby preventing chitin-triggered immunity [35,36].

In a forward genetic screen for *C. higginsianum* virulence on *A. thaliana* via *Agrobacterium tumefaciens*-mediated transformation (ATMT)-based insertional mutagenesis, Plaumann and colleagues [11] identified two T-DNA insertion mutants, vir-49 and vir-51, that lacked Chr11. Both mutants exhibited a decreased level of virulence on *A*. *thaliana* Col-0 due to an inefficient shift from biotrophy to necrotrophy, evidenced by less than 3% of the primary hyphae generating secondary hyphae in the infected cells. However, the lack of the chromosome did not impact vegetative fungal growth or conidiation, indicating that Chr11 is a conditionally dispensable minichromosome.

## 5. Perspectives

With the inception of third-generation sequencing and the subsequent correction of the resulting long reads using second-generation sequencing, it has become possible to generate gapless fungal genome assemblies at the chromosome level, enabling the identification of minichromosomes. Minichromosomes in the hemibiotrophic *Colletotrichum* pathogens appear to be strain-specific innovations as the strains within species, let alone between species, do not share minichromosomes. In addition, they are the gene-sparse regions of the genomes and are not enriched with genes known to be implicated in fungal virulence/pathogenicity on hosts, including but not limited to effectors, CAZymes and secondary metabolism genes. The minichromosomes, especially in the *C. graminicola* strains, are highly compartmentalized into AT-rich and GC-rich blocks, with the former occupying the majority of the chromosomes, and hence contain a higher content of repetitive DNA elements and lower gene contents. The three *Colletotrichum* pathogens that we discussed carry a single conditionally dispensable minichromosome, which, especially in *C. graminicola* and *C. lentis*, are transcriptionally less active than the core chromosomes. The limited number of transcriptionally active genes in the conditionally dispensable minichromosomes Cg-Chr12 and Cl-Chr12 located in the GC-rich blocks that are more protected from erosion by RIPMs than Ch-Chr11 might be an adaptation, restricting *C. graminicola* and *C. lentis* to infect a single host species, as opposed *C. higginsianum*, which can invade multiple species of crucifers. It would be interesting to assess the role of minichromosomes in the adaptation to various stresses, e.g., osmotic, oxidative and salt stresses, and cell wall damage, enabling fungal pathogens to invade and successfully colonize host species.

## Figures and Tables

**Table 1:** General features of minichromosomes in four strains of three hemibiotrophic *Colletotrichum* pathogens and features thereof.

Strains	Features	Chr11	Chr12	Chr13
T1-3-3	Length (bp)	714,004	618,571	561,802
	GC content (%)	29.10	31.24	30.74
	Repetitive DNA (%)	11.14	11.11	12.13
	RIP-affected genomic proportion (%)	80.97	71.65	75.36
	Gene models	32	31	26
	RNA-Seq evidence (%)	81.25	58.06	76.92
	Effector candidates	0	1	0
	CAZymes	0	0	0
	SMBSE ^a^	0	0	0
	Conditionally dispensability ^b^	No	Yes	No
M1.001	Length (bp)	729,294	559,114	550,543
	GC content (%)	29.90	30.10	30.31
	Repetitive DNA (%)	10.59	10.78	8.86
	RIP-affected genomic proportion (%)	77.38	74.44	75.68
	Gene models	43	43	35
	RNA-Seq evidence (%)	65	58	43
	Effector candidates	0	0	0
	CAZymes	0	0	0
	SMBSE	0	0	0
	Conditionally dispensability	Unknown	Unknown	Unknown
CT-30	Length (bp)	1,517,430	386,020	-
	GC content (%)	37.57	37.26	-
	Repetitive DNA (%)	9.21	8.02	-
	RIP-affected genomic proportion (%)	63.82	60.41	-
	Gene models	164	38	-
	RNA-Seq evidence (%)	na ^d^	na ^d^	-
	Effector candidates	9	0	-
	CAZymes	0	0	-
	SMBSE	5	7	-
	Conditionally dispensability ^c^	Yes	No	-
IMI 349063A	Length (bp)	646,208	597,935	-
	GC content (%)	49.35	47.20	-
	Repetitive DNA (%)	13.65	8.89	-
	RIP-affected genomic proportion (%)	8.89	15.38	-
	Gene models	138	133	-
	RNA-Seq evidence (%)	na ^d^	na ^d^	-
	Effector candidates	10	7	-
	CAZymes	1	1	-
	SMBSE	0	1	-
	Conditionally dispensability ^b^	Yes	No	-

^a^ Secondary metabolite backbone synthesis enzymes; ^b^ molecular evidence; ^c^ genetic evidence; ^d^ not available.

## Data Availability

The data that support the findings of this study are available from the corresponding author upon reasonable request.

## Supplementary Materials

The following supporting information can be downloaded at https://www.mdpi.com/article/10.3390/ijms25010198/s1.
