# Supplementary material for: The Hidden Truths of Fungal Virulence and Adaptation on Hosts: Unraveling the Conditional Dispensability of Minichromosomes in the Hemibiotrophic Colletotrichum Pathogens
Source: Int J Mol Sci. 2023 Dec 22;25(1):198. doi: 10.3390/ijms25010198 (PMC10779208; doi:10.3390/ijms25010198)
Supplement: Supplementary file 1 [file ijms-25-00198-s001.zip › ijms-2761866-supplementary.pdf]

Supplementary Table S1. The homologous genes in Chr11 of the *Colletotrichum graminicola* strains T1-3-3 and M1.001

T1-3-3-Chr11	M1.001-Chr11	Functional annotation of the encoded proteins
GME6610_g	–	Hypothetical protein
GME6611_g	*CGRA01v4_15054*	Hypothetical protein
GME6612_g	*CGRA01v4_15053*	Hypothetical protein
GME6613_g	*CGRA01v4_15043*	Hypothetical protein
GME6614_g	*CGRA01v4_15042*	Hypothetical protein
GME6615_g	*CGRA01v4_15041*	Hypothetical protein
GME6616_g	*CGRA01v4_15040*	Hypothetical protein
GME6617_g	*CGRA01v4_15039*	Hypothetical protein
GME6618_g	*CGRA01v4_15038*	Hypothetical protein
GME6619_g	*CGRA01v4_15035*	Ulp1 protease family protein
GME6620_g	–	Ulp1 protease family protein
GME6621_g	*CGRA01v4_15034*	Hypothetical protein
GME6622_g	–	Exo-1,3-beta-D-glucanase
GME6623_g	*CGRA01v4_15033*	Hypothetical protein
GME6624_g	*CGRA01v4_15031*	Hypothetical protein
GME6625_g	*CGRA01v4_15030*	Hypothetical protein
GME6626_g	–	Hypothetical protein
GME6627_g	*CGRA01v4_15028*	Hypothetical protein
GME6628_g	–	Hypothetical protein
GME6629_g	*CGRA01v4_15025*	Hypothetical protein
GME6630_g	*CGRA01v4_15024*	Hypothetical protein
GME6631_g	*CGRA01v4_15023*	Hypothetical protein
GME6632_g	*CGRA01v4_15022*	Hypothetical protein
GME6633_g	*CGRA01v4_15020*	Hypothetical protein
GME6634_g	*CGRA01v4_15019*	Hypothetical protein
GME6635_g	*CGRA01v4_15018*	Hypothetical protein
GME6636_g	*CGRA01v4_15017*	Hypothetical protein
GME6637_g	*CGRA01v4_15016*	Hypothetical protein
GME6638_g	*CGRA01v4_15015*	Hypothetical protein
GME6639_g	–	Unique to T1-3-3
GME6640_g	–	Hypothetical protein
GME6641_g	*CGRA01v4_15013*	Hypothetical protein
–	*CGRA01v4_15012*	Hypothetical protein
–	*CGRA01v4_15014*	Unique to M1.001
–	*CGRA01v4_15021*	Hypothetical protein
–	*CGRA01v4_15026*	Unique to M1.001
–	*CGRA01v4_15027*	Hypothetical protein

–	*CGRA01v4_15029*	Hypothetical protein
–	*CGRA01v4_15032*	Ankyrin unc44
–	*CGRA01v4_15036*	Hypothetical protein
–	*CGRA01v4_15037*	Hypothetical protein
–	*CGRA01v4_15044*	Hypothetical protein
–	*CGRA01v4_15045*	Hypothetical protein
–	*CGRA01v4_15046*	Hypothetical protein
–	*CGRA01v4_15047*	Hypothetical protein
–	*CGRA01v4_15048*	Hypothetical protein
–	*CGRA01v4_15049*	Hypothetical protein
–	*CGRA01v4_15050*	Hypothetical protein
–	*CGRA01v4_15051*	Hypothetical protein
–	*CGRA01v4_15052*	Hypothetical protein

Supplementary Table S2. miRNAs in the *Colletotrichum graminicola* strain T1-3-3

Minichromosome	Start	End	Strand	miRNA type
Chr11	148195	148235	-	mir-684
Chr11	148268	148283	-	mir-684
Chr11	361359	361426	-	mir-598
Chr11	361350	361423	+	mir-598
Chr11	432312	432416	+	mir-598
Chr11	432321	432425	-	mir-598
Chr11	585324	585367	-	mir-684
Chr12	391589	391647	-	mir-598
Chr12	391586	391641	+	mir-598
Chr12	392378	392459	+	mir-598
Chr12	91121	91161	-	mir-598
Chr13	402923	402978	-	mir-598
Chr13	429667	429702	+	mir-598
Chr13	544385	544423	-	mir-598
Chr13	544388	544429	+	mir-598

Supplementary Table S3. Genes located in Chr12 of the *Colletotrichum graminicola* strain T1-3-3 (adapted from Ma et al. 2023)

Gene ID	Functional annotation of the encoded proteins
GME10836	Hypothetical protein
GME10837	Hypothetical protein
GME10838	Hypothetical protein
GME10839	Hypothetical protein
GME10840	Unique to T1-3-3
GME10841	Hypothetical protein
GME10842	Hypothetical protein
GME10843	Unique to T1-3-3
GME10844	Unique to T1-3-3
GME10845	Hypothetical protein
GME10846	Hypothetical protein
GME10847	Hypothetical protein
GME10848	Hypothetical protein
GME10849	Unique to T1-3-3
GME10850	Hypothetical protein
GME10851	Unique to T1-3-3
GME10852	Unique to T1-3-3
GME10853	Hypothetical protein
GME10854	Hypothetical protein
GME10855	Hypothetical protein
GME10856	Hypothetical protein
GME10857	Hypothetical protein
GME10858	Hypothetical protein
GME10859	Hypothetical protein
GME10860	Hypothetical protein
GME10861	Hypothetical protein
GME10862	LPXTG-domain-containing protein
GME10863	Unique to T1-3-3
GME10864	Unique to T1-3-3
GME10865	Hypothetical protein
GME10866	Hypothetical protein

Supplementary Table S4. Genes located in Chr12 of the *Colletotrichum graminicola* strain M1.001 (adapted from Becerra et al. 2023)

Gene ID	Functional annotation of the encoded proteins
CGRA01v4_15055	Hypothetical protein
CGRA01v4_15056	Sentrin/sumo-specific protease
CGRA01v4_15057	Hypothetical protein
CGRA01v4_15058	Hypothetical protein
CGRA01v4_15059	Hypothetical protein
CGRA01v4_15060	Unique to M1.001
CGRA01v4_15061	Hypothetical protein
CGRA01v4_15062	Hypothetical protein
CGRA01v4_15063	Hypothetical protein
CGRA01v4_15064	Hypothetical protein
CGRA01v4_15065	Hypothetical protein
CGRA01v4_15066	Hypothetical protein
CGRA01v4_15067	Hypothetical protein
CGRA01v4_15068	Hypothetical protein
CGRA01v4_15069	Hypothetical protein
CGRA01v4_15070	Unique to M1.001
CGRA01v4_15071	Unique to M1.001
CGRA01v4_15072	Hypothetical protein
CGRA01v4_15073	Hypothetical protein
CGRA01v4_15074	Hypothetical protein
CGRA01v4_15075	Unique to M1.001
CGRA01v4_15076	Hypothetical protein
CGRA01v4_15077	Hypothetical protein
CGRA01v4_15078	Hypothetical protein
CGRA01v4_15079	Hypothetical protein
CGRA01v4_15080	Hypothetical protein
CGRA01v4_15081	Unique to M1.001
CGRA01v4_15082	Hypothetical protein
CGRA01v4_15083	Linoleate diol synthase
CGRA01v4_15084	Chloroperoxidase
CGRA01v4_15085	VID27 cytoplasmic protein
CGRA01v4_15086	Hypothetical protein
CGRA01v4_15087	Hypothetical protein
CGRA01v4_15088	Hypothetical protein
CGRA01v4_15089	Hypothetical protein
CGRA01v4_15090	Hypothetical protein
CGRA01v4_15091	Hypothetical protein
CGRA01v4_15092	Unique to M1.001
CGRA01v4_15093	Hypothetical protein
CGRA01v4_15094	Hypothetical protein

CGRA01v4_15095	Ulp1 protease family protein
CGRA01v4_15096	BRO1-like domain-containing protein
CGRA01v4_15097	Methyltransferase type 11

Supplementary Table S5. Genes located in Chr13 of the *Colletotrichum graminicola* strain T1-3-3 (adapted from Ma et al. 2023)

Gene ID	Functional annotation of the encoded proteins
GME10810	Hypothetical protein
GME10811	Unique to T1-3-3
GME10812	Unique to T1-3-3
GME10813	Unique to T1-3-3
GME10814	Hypothetical protein
GME10815	Hypothetical protein
GME10816	Hypothetical protein
GME10817	Hypothetical protein
GME10818	Unique to T1-3-3
GME10819	Unique to T1-3-3
GME10820	Unique to T1-3-3
GME10821	Hypothetical protein
GME10822	Hypothetical protein
GME10823	Hypothetical protein
GME10824	Hypothetical protein
GME10825	Hypothetical protein
GME10826	Hypothetical protein
GME10827	Unique to T1-3-3
GME10828	Hypothetical protein
GME10829	Hypothetical protein
GME10830	Low-quality protein
GME10831	Hypothetical protein
GME10832	Unique to T1-3-3
GME10833	Hypothetical protein
GME10834	Unique to T1-3-3
GME10835	Unique to T1-3-3

Supplementary Table S6. Genes located in Chr13 of the *Colletotrichum graminicola* strain M1.001 (adapted from Becerra et al. 2023)

Gene ID	Functional annotation of the encoded proteins
CGRA01v4_15098	Hypothetical protein
CGRA01v4_15099	Unique to M1.001
CGRA01v4_15100	Hypothetical protein
CGRA01v4_15101	Hypothetical protein
CGRA01v4_15102	Unique to M1.001
CGRA01v4_15103	Hypothetical protein
CGRA01v4_15104	Hypothetical protein
CGRA01v4_15105	Hypothetical protein
CGRA01v4_15106	Hypothetical protein
CGRA01v4_15107	Unique to M1.001
CGRA01v4_15108	Hypothetical protein
CGRA01v4_15109	Hypothetical protein
CGRA01v4_15110	Hypothetical protein
CGRA01v4_15111	Hypothetical protein
CGRA01v4_15113	Hypothetical protein
CGRA01v4_15114	Hypothetical protein
CGRA01v4_15115	Hypothetical protein
CGRA01v4_15116	Unique to M1.001
CGRA01v4_15117	Hypothetical protein
CGRA01v4_15118	Hypothetical protein
CGRA01v4_15119	Hypothetical protein
CGRA01v4_15120	Hypothetical protein
CGRA01v4_15121	Unique to M1.001
CGRA01v4_15122	Hypothetical protein
CGRA01v4_15123	Hypothetical protein
CGRA01v4_15124	Hypothetical protein
CGRA01v4_15125	Hypothetical protein
CGRA01v4_15126	Hypothetical protein
CGRA01v4_15127	Hypothetical protein
CGRA01v4_15128	Hypothetical protein
CGRA01v4_15129	Hypothetical protein
CGRA01v4_15130	Hypothetical protein
CGRA01v4_15131	Hypothetical protein
CGRA01v4_15132	Unique to M1.001
CGRA01v4_15133	Hypothetical protein

Supplementary Table S7. Genes located in Chr11 of the *Colletotrichum lentis* strain CT-30 (adapted from Bhadauria et al. 2019)

Gene ID	Functional annotation of the encoded proteins
scaffold14-1	P450 monooxygenase
scaffold14-2	Zn(2)-C6 fungal-type domain-containing protein
scaffold14-3	Unique to CT-30
scaffold14-4	NUDIX domain-containing protein
scaffold14-5	glycoside hydrolase family 18 protein
scaffold14-6	LysM domain-containing protein
scaffold14-7	galactonate dehydratase
scaffold14-8	Thiol-disulfide exchange intermediate
scaffold14-9	hypothetical protein
scaffold14-10	4-coumarate-CoA ligase
scaffold14-11	hypothetical protein
scaffold14-12	Ank-repeat protein mbp1
scaffold14-13	hypothetical protein
scaffold14-14	Alternative oxidase
scaffold14-15	Unique to CT-30
scaffold14-16	CFEM domain-containing protein
scaffold14-17	heterokaryon incompatibility protein
scaffold14-18	hypothetical protein
scaffold14-19	BTB/POZ domain-containing protein
scaffold14-20	hypothetical protein
scaffold14-21	hypothetical protein
scaffold14-22	hypothetical protein
scaffold14-23	Unique to CT-30
scaffold14-24	hypothetical protein
scaffold14-25	hypothetical protein
scaffold14-26	Tryptophan dimethylallyltransferase
scaffold14-27	glycoside hydrolase family 18 protein
scaffold14-28	WSC domain protein
scaffold14-29	WD domain-containing protein
scaffold14-30	secreted protein
scaffold14-31	alkaline phosphatase-like protein
scaffold14-32	effector protein PevD1
scaffold14-33	hypothetical protein
scaffold14-34	Unique to CT-30
scaffold14-35	hypothetical protein
scaffold14-36	Unique to CT-30
scaffold14-37	collagen triple helix repeat-containing protein
scaffold14-38	FAD binding domain-containing protein
scaffold14-39	Short-chain dehydrogenase chyC
scaffold14-40	Amidase chyE

scaffold14-41	NRPS
scaffold14-42	NRPS
scaffold14-43	Glutamine amidotransferase class-I
scaffold14-44	Unique to CT-30
scaffold14-45	Unique to CT-30
scaffold14-46	Unique to CT-30
scaffold14-47	Unique to CT-30
scaffold14-48	Unique to CT-30
scaffold14-49	hypothetical protein
scaffold14-50	hypothetical protein
scaffold14-51	Pep1
scaffold14-52	hypothetical protein
scaffold14-53	Unique to CT-30
scaffold14-54	Unique to CT-30
scaffold14-55	hypothetical protein
scaffold14-56	Subtilisin-like protease 2
scaffold14-57	Unique to CT-30
scaffold14-58	Unique to CT-30
scaffold14-59	beta-1,3-glucosidase
scaffold14-60	Unique to CT-30
scaffold14-61	Unique to CT-30
scaffold14-62	Acid trehalase
scaffold14-63	ef-hand calcium-binding domain-containing protein
scaffold14-64	cytochrome P450
scaffold14-65	lovastatin nonaketide synthase
scaffold14-66	Unique to CT-30
scaffold14-67	Unique to CT-30
scaffold14-68	serine threonine protein kinase
scaffold14-69	Unique to CT-30
scaffold14-70	Unique to CT-30
scaffold14-71	Unique to CT-30
scaffold14-72	Unique to CT-30
scaffold14-73	serine/threonine protein kinase
scaffold14-74	acyl-coenzyme A:6-aminopenicillanic-acid-acyltransferase form
scaffold14-75	putative bifunctional amine oxidase
scaffold14-76	hypothetical protein
scaffold14-77	Killer toxin subunits alpha/beta
scaffold14-78	Unique to CT-30
scaffold14-79	hypothetical protein
scaffold14-80	putative cholera enterotoxin subunit A2
scaffold14-81	Vegetative incompatibility protein HET-E-1
scaffold14-82	Unique to CT-30
scaffold14-83	carbon-nitrogen hydrolase

scaffold14-84	NACHT and WD domain protein
scaffold14-85	splicing factor 3A subunit 2-like
scaffold14-86	kinase-like domain-containing protein
scaffold14-87	mitochondrial chaperone bcs1
scaffold14-88	Unique to CT-30
scaffold14-89	putative proline-rich protein 21
scaffold14-90	WD40 repeat-like protein
scaffold14-91	Protein-ribulosamine 3-kinase
scaffold14-92	Unique to CT-30
scaffold14-93	putative RNA-directed DNA polymerase from transposon BS
scaffold14-94	reverse transcriptase
scaffold14-95	integral membrane protein
scaffold14-96	RNA-directed DNA polymerase from mobile element jockey
scaffold14-97	hypothetical protein
scaffold14-98	bZIP family transcription factor
scaffold14-99	Cytoplasmic protein
scaffold14-100	Alcohol dehydrogenase
scaffold14-101	C6 zinc finger domain-containing protein
scaffold14-102	reverse transcriptase RNaseH
scaffold14-103	Unique to CT-30
scaffold14-104	histidine kinase
scaffold14-105	Unique to CT-30
scaffold14-106	putative RNA-directed DNA polymerase from transposon BS
scaffold14-107	basic region leucine zipper
scaffold14-108	transcription factor bZIP protein
scaffold14-109	tpr domain protein
scaffold14-110	putative chromo domain-containing protein
scaffold14-111	G protein-coupled glucose receptor regulating Gpa2-domain-containing protein
scaffold14-112	Unique to CT-30
scaffold14-113	hypothetical protein
scaffold14-114	Unique to CT-30
scaffold14-115	isoform 2 of serine/threonine-protein phosphatase 6 regulatory ankyrin repeat subunit A
scaffold14-116	putative glycosyl hydrolase family 18
scaffold14-117	hypothetical protein
scaffold14-118	hypothetical protein
scaffold14-119	Unique to CT-30
scaffold14-120	Unique to CT-30
scaffold14-121	Unique to CT-30
scaffold14-122	Sentrin sumo-specific
scaffold14-123	Unique to CT-30
scaffold14-124	hypothetical protein

scaffold14-125	hypothetical protein
scaffold14-126	hypothetical protein
scaffold14-127	hypothetical protein
scaffold14-128	L-aminoadipate-semialdehyde dehydrogenase
scaffold14-129	Alcohol dehydrogenase 2
scaffold14-130	Nucleoside-diphosphate-sugar epimerase
scaffold14-131	MFS transporter
scaffold14-132	Maltose O-acetyltransferase
scaffold14-133	phosphoglycerate mutase-like protein
scaffold14-134	N-acetylglucosamine-induced protein 1
scaffold14-135	Tdp-4-oxo-6-deoxy-d-glucose transaminase
scaffold14-136	Carboxylic ester hydrolase
scaffold14-137	hypothetical protein
scaffold14-138	hypothetical protein
scaffold14-139	Unique to CT-30
scaffold14-140	Unique to CT-30
scaffold14-141	hypothetical protein
scaffold14-142	hypothetical protein
scaffold14-143	hypothetical protein
scaffold14-144	Transposon Tf2-9 polyprotein
scaffold14-145	hypothetical protein
scaffold14-146	Pisatin demethylase PDA1
scaffold14-147	O-methyltransferase
scaffold14-148	NRPS
scaffold14-149	NRPS
scaffold14-150	hypothetical protein
scaffold14-151	Unique to CT-30
scaffold14-152	hypothetical protein
scaffold14-153	Unique to CT-30
scaffold14-154	hypothetical protein
scaffold14-155	hypothetical protein
scaffold14-156	hypothetical protein
scaffold14-157	hypothetical protein
scaffold14-158	hypothetical protein
scaffold14-159	hypothetical protein
scaffold14-160	Unique to CT-30
scaffold14-161	hypothetical protein
scaffold14-162	putative translation initiation factor IF-2
scaffold14-163	Di-copper centre-containing protein
scaffold14-164	hypothetical protein

Supplementary Table S8. Genes located in Chr12 of the *Colletotrichum lentis* strain CT-30 (adapted from Bhadauria et al. 2019)

Gene ID	Functional annotation of the encoded proteins
scaffold20-1	hypothetical protein
scaffold20-2	ankyrin
scaffold20-3	hypothetical protein
scaffold20-4	Unique to CT-30
scaffold20-5	hypothetical protein
scaffold20-6	PKS
scaffold20-7	Unique to CT-30
scaffold20-8	Unique to CT-30
scaffold20-9	NRPS
scaffold20-10	NRPS
scaffold20-11	NRPS
scaffold20-12	NRPS
scaffold20-13	NRPS
scaffold20-14	NRPS
scaffold20-15	hypothetical protein
scaffold20-16	hypothetical protein
scaffold20-17	Unique to CT-30
scaffold20-18	Unique to CT-30
scaffold20-19	hypothetical protein
scaffold20-20	hypothetical protein
scaffold20-21	Unique to CT-30
scaffold20-22	hypothetical protein
scaffold20-23	hypothetical protein
scaffold20-24	Ulp1
scaffold20-25	hypothetical protein
scaffold20-26	Unique to CT-30
scaffold20-27	hypothetical protein
scaffold20-28	hypothetical protein
scaffold20-29	hypothetical protein
scaffold20-30	Unique to CT-30
scaffold20-31	hypothetical protein
scaffold20-32	NB-ARC domain-containing protein
scaffold20-33	serine/threonine-protein kinase
scaffold20-34	DNA repair helicase
scaffold20-35	Unique to CT-30
scaffold20-36	conserved glycine-rich protein
scaffold20-37	Unique to CT-30
scaffold20-38	hypothetical protein

Supplementary Table S9. Genes located in Chr11 of the *Colletotrichum higginsianum* strain IMI 349063A (adapted from Dallery et al. 2017)

Gene ID	Functional annotation of the encoded proteins
CH63R_14381	Nudix domain-containing protein
CH63R_14382	hypothetical protein
CH63R_14383	EC7 protein
CH63R_14384	hypothetical protein
CH63R_14385	hypothetical protein
CH63R_14386	hypothetical protein
CH63R_14387	Phospholipase Carboxylesterase
CH63R_14388	hypothetical protein
CH63R_14389	EC12a protein
CH63R_14390	Nudix domain-containing protein
CH63R_14391	Aurora kinase 2 splicing
CH63R_14392	Integral membrane protein
CH63R_14393	Nudix domain-containing protein
CH63R_14394	hypothetical protein
CH63R_14395	protease
CH63R_14396	hypothetical protein
CH63R_14397	ATP-dependent DNA helicase PIF1
CH63R_14398	hypothetical protein
CH63R_14399	ankyrin repeat protein
CH63R_14400	hypothetical protein
CH63R_14401	hypothetical protein
CH63R_14402	von Willebrand factor
CH63R_14403	von Willebrand factor
CH63R_14404	hypothetical protein
CH63R_14405	Tata-box-binding protein
CH63R_14406	Endochitinase
CH63R_14407	kinesin
CH63R_14408	hypothetical protein
CH63R_14409	hypothetical protein
CH63R_14410	Ank-repeat protein mbp1
CH63R_14411	F-box domain-containing protein
CH63R_14412	Duf323 domain-containing protein
CH63R_14413	hypothetical protein
CH63R_14414	Ankyrin repeat protein
CH63R_14415	hypothetical protein
CH63R_14416	hypothetical protein
CH63R_14417	hypothetical protein
CH63R_14418	Peptidase C14
CH63R_14419	hypothetical protein
CH63R_14420	hypothetical protein

CH63R_14421	hypothetical protein
CH63R_14422	hypothetical protein
CH63R_14423	Kinesin light
CH63R_14424	hypothetical protein
CH63R_14425	Glycosyl transferase
CH63R_14426	hypothetical protein
CH63R_14427	Ank-repeat protein mbp1
CH63R_14428	Pfs domain-containing protein
CH63R_14429	hypothetical protein
CH63R_14430	hypothetical protein
CH63R_14431	hypothetical protein
CH63R_14432	hypothetical protein
CH63R_14433	hypothetical protein
CH63R_14434	hypothetical protein
CH63R_14435	hypothetical protein
CH63R_14436	hypothetical protein
CH63R_14437	hypothetical protein
CH63R_14438	Phosphorylase superfamily protein
CH63R_14439	NUDIX domain-containing protein
CH63R_14440	reverse transcriptase and RNase H
CH63R_14441	Serine threonine protein kinase
CH63R_14442	ATP-dependent DNA helicase
CH63R_14443	hypothetical protein
CH63R_14444	Ebs-bah-phd domain-containing protein
CH63R_14445	WD domain-containing protein
CH63R_14446	hypothetical protein
CH63R_14447	High-affinity methionine permease
CH63R_14448	hypothetical protein
CH63R_14449	hypothetical protein
CH63R_14450	hypothetical protein
CH63R_14451	hypothetical protein
CH63R_14452	hypothetical protein
CH63R_14453	hypothetical protein
CH63R_14454	hypothetical protein
CH63R_14455	DNA repair helicase
CH63R_14456	hypothetical protein
CH63R_14457	hypothetical protein
CH63R_14458	hypothetical protein
CH63R_14459	hypothetical protein
CH63R_14460	Aurora kinase 2 splicing
CH63R_14461	hypothetical protein
CH63R_14462	hypothetical protein
CH63R_14463	Tetratricopeptide repeat domain containing protein

CH63R_14464	hypothetical protein
CH63R_14465	hypothetical protein
CH63R_14466	hypothetical protein
CH63R_14467	hypothetical protein
CH63R_14468	hypothetical protein
CH63R_14469	Hst3 protein
CH63R_14470	hypothetical protein
CH63R_14471	Peptidase C14
CH63R_14472	Peptidase C14
CH63R_14473	hypothetical protein
CH63R_14474	hypothetical protein
CH63R_14475	Integral membrane protein
CH63R_14476	reverse transcriptase and RNase H
CH63R_14477	hypothetical protein
CH63R_14478	Ulp1 protease family protein
CH63R_14479	TPR domain-containing protein
CH63R_14480	hypothetical protein
CH63R_14481	hypothetical protein
CH63R_14482	hypothetical protein
CH63R_14483	C2H2 finger domain-containing protein
CH63R_14484	WD domain-containing protein
CH63R_14485	hypothetical protein
CH63R_14486	hypothetical protein
CH63R_14487	Ulp1 protease family protein
CH63R_14488	hypothetical protein
CH63R_14489	hypothetical protein
CH63R_14490	hypothetical protein
CH63R_14491	TAM domain methyltransferase
CH63R_14492	glycosyl hydrolase family 18
CH63R_14493	C-x8-C-x5-C-x3-H type zinc finger protein
CH63R_14494	hypothetical protein
CH63R_14495	hypothetical protein
CH63R_14496	chromo domain-containing protein
CH63R_14497	hypothetical protein
CH63R_14498	hypothetical protein
CH63R_14499	hypothetical protein
CH63R_14500	Ankyrin repeat domain-containing protein 52
CH63R_14501	hypothetical protein
CH63R_14502	hypothetical protein
CH63R_14503	hypothetical protein
CH63R_14504	hypothetical protein
CH63R_14505	Zn 2cys6 transcription factor
CH63R_14506	Zn 2cys6 transcription factor

CH63R_14507	EC21 protein
CH63R_14508	Beta-1,4-glucosidase
CH63R_14509	EC7 protein
CH63R_14510	hypothetical protein
CH63R_14511	Nudix domain-containing protein
CH63R_14512	Alternative oxidase
CH63R_14513	Carboxypeptidase S1
CH63R_14514	hypothetical protein
CH63R_14515	hypothetical protein
CH63R_14516	EC12a protein
CH63R_14517	24 kDa metalloproteinase
CH63R_14518	hypothetical protein

Supplementary Table S10. Genes located in Chr12 of the *Colletotrichum higginsianum* strain IMI 349063A (adapted from Dallery et al. 2017)

**Gene ID**	**Functional annotation of the encoded proteins**
CH63R_14519	Acid trehalase
CH63R_14520	Duf341 family
CH63R_14521	cytochrome P450
CH63R_14522	Beta-ketoacyl synthase domain-containing protein
CH63R_14523	Nacht and ankyrin domain protein
CH63R_14524	hypothetical protein
CH63R_14525	hypothetical protein
CH63R_14526	hypothetical protein
CH63R_14527	hypothetical protein
CH63R_14528	hypothetical protein
CH63R_14529	hypothetical protein
CH63R_14530	hypothetical protein
CH63R_14531	Ulp1 protease family protein
CH63R_14532	hypothetical protein
CH63R_14533	hypothetical protein
CH63R_14534	Chitinase
CH63R_14535	LysM domain-containing protein
CH63R_14536	hypothetical protein
CH63R_14537	hypothetical protein
CH63R_14538	hypothetical protein
CH63R_14539	zinc knuckle
CH63R_14540	hypothetical protein
CH63R_14541	hypothetical protein
CH63R_14542	hypothetical protein
CH63R_14543	hypothetical protein
CH63R_14544	hypothetical protein
CH63R_14545	hypothetical protein
CH63R_14546	hypothetical protein
CH63R_14547	hypothetical protein
CH63R_14548	Sentrin sumo-specific
CH63R_14549	hypothetical protein
CH63R_14550	hypothetical protein
CH63R_14551	hypothetical protein
CH63R_14552	hypothetical protein
CH63R_14553	hypothetical protein
CH63R_14554	hypothetical protein
CH63R_14555	hypothetical protein
CH63R_14556	hypothetical protein
CH63R_14557	PKS-NRPS hybrid

CH63R_14558	hypothetical protein
CH63R_14559	hypothetical protein
CH63R_14560	hypothetical protein
CH63R_14561	hypothetical protein
CH63R_14562	hypothetical protein
CH63R_14563	Tyrosinase
CH63R_14564	hypothetical protein
CH63R_14565	Metalloprotease
CH63R_14566	hypothetical protein
CH63R_14567	Glycosyl hydrolase family 92
CH63R_14568	hypothetical protein
CH63R_14569	hypothetical protein
CH63R_14570	hypothetical protein
CH63R_14571	hypothetical protein
CH63R_14572	hypothetical protein
CH63R_14573	hypothetical protein
CH63R_14574	hypothetical protein
CH63R_14575	hypothetical protein
CH63R_14576	hypothetical protein
CH63R_14577	hypothetical protein
CH63R_14578	hypothetical protein
CH63R_14579	hypothetical protein
CH63R_14580	hypothetical protein
CH63R_14581	hypothetical protein
CH63R_14582	hypothetical protein
CH63R_14583	Membrane protein
CH63R_14584	Gag protein
CH63R_14585	hypothetical protein
CH63R_14586	hypothetical protein
CH63R_14587	Glutathione-dependent formaldehyde-activating enzyme
CH63R_14588	hypothetical protein
CH63R_14589	hypothetical protein
CH63R_14590	hypothetical protein
CH63R_14591	hypothetical protein
CH63R_14592	hypothetical protein
CH63R_14593	hypothetical protein
CH63R_14594	hypothetical protein
CH63R_14595	hypothetical protein
CH63R_14596	hypothetical protein
CH63R_14597	hypothetical protein
CH63R_14598	hypothetical protein
CH63R_14599	hypothetical protein
CH63R_14600	Ulp1 protease family protein

CH63R_14601	hypothetical protein
CH63R_14602	hypothetical protein
CH63R_14603	DNA polymerase pol2
CH63R_14604	hypothetical protein
CH63R_14605	hypothetical protein
CH63R_14606	hypothetical protein
CH63R_14607	DNA-directed RNA polymerase
CH63R_14608	hypothetical protein
CH63R_14609	hypothetical protein
CH63R_14610	Cytochrome P450
CH63R_14611	cytochrome P450
CH63R_14612	Potassium transport protein 1
CH63R_14613	hypothetical protein
CH63R_14614	Homoserine acetyltransferase
CH63R_14615	MFS monocarboxylate transporter
CH63R_14616	Riboflavin transporter MCH5
CH63R_14617	endoribonuclease L-PSP
CH63R_14618	Fungal specific transcription factor
CH63R_14619	hypothetical protein
CH63R_14620	hypothetical protein
CH63R_14621	hypothetical protein
CH63R_14622	chromo domain-containing protein
CH63R_14623	hypothetical protein
CH63R_14624	transposase
CH63R_14625	hypothetical protein
CH63R_14626	hypothetical protein
CH63R_14627	hypothetical protein
CH63R_14628	hypothetical protein
CH63R_14629	hypothetical protein
CH63R_14630	hypothetical protein
CH63R_14631	hypothetical protein
CH63R_14632	hypothetical protein
CH63R_14633	hypothetical protein
CH63R_14634	hypothetical protein
CH63R_14635	hypothetical protein
CH63R_14636	hypothetical protein
CH63R_14637	hypothetical protein
CH63R_14638	hypothetical protein
CH63R_14639	hypothetical protein
CH63R_14640	hypothetical protein
CH63R_14641	hypothetical protein
CH63R_14642	Sentrin sumo-specific
CH63R_14643	hypothetical protein

CH63R_14644	hypothetical protein
CH63R_14645	Cap22 protein
CH63R_14646	hypothetical protein
CH63R_14647	Ank-repeat protein mbp1
CH63R_14648	EC51a protein
CH63R_14649	UDP-N-acetylglucosamine transferase subunit ALG13
CH63R_14650	hypothetical protein
CH63R_14651	hypothetical protein
